# Hypoxia induced lipid droplet accumulation promotes resistance to ferroptosis in prostate cancer

**DOI:** 10.18632/oncotarget.28750

**Published:** 2025-06-25

**Authors:** Shailender S. Chauhan, Andres D. Vizzerra, Hope Liou, Caitlyn E. Flores, Ashley J. Snider, Justin M. Snider, Noel A. Warfel

**Affiliations:** ^1^Cellular and Molecular Medicine, University of Arizona, Tucson, AZ 85724, USA; ^2^Cancer Biology Graduate Program, University of Arizona, Tucson, AZ 85721, USA; ^3^School of Nutritional Sciences and Wellness, University of Arizona, Tucson, AZ 85721, USA; ^4^University of Arizona Cancer Center, University of Arizona, Tucson, AZ 85724, USA

**Keywords:** hypoxia, lipid droplets, ferroptosis, resistance, prostate

## Abstract

Ferroptosis is a mode of cell death that relies on iron metabolism and lipid peroxidation. Preclinical and clinical studies indicate that ferroptosis suppresses tumor growth, and dysregulation of ferroptosis promotes treatment resistance in cancer. Hypoxia is a universal feature of solid tumors that is particularly relevant to prostate cancer (PCa), which arises in the hypoxic peripheral zone of the organ. Hypoxia has been implicated in resistance to ferroptosis and other forms of cell death, but how hypoxia impacts the sensitivity of PCa to ferroptosis inducing agents (FINs) has not been well studied. Here, we show that hypoxia dramatically reduces the sensitivity of PCa cell lines to mechanistically distinct FINs, Erastin (xCT inhibitor) and RLS3 (GPX4 inhibitor) by inducing lipid droplet (LD) accumulation. Transcriptomic analysis revealed that hypoxia significantly reduced the expression of genes related to incorporating polyunsaturated fatty acids into phospholipids (ACSL4, LPCAT3), and parallel lipidomic analysis demonstrated that hypoxia significantly decreased the levels of the ferroptosis-prone lipid class, phosphatidylethanolamine (PE) and increased production of neutral lipid species, cholesteryl ester (ChE (22:5)) and triglycerides (TG(48:1), TG:(50:4), and TG(58:4)). Targeting LD biogenesis and *de novo* lipogenesis did not alter sensitivity to RSL3 under hypoxia. These findings suggest that hypoxia promotes ferroptosis resistance in PCa by altering lipid metabolism at the transcriptional level, by producing lipids that are less susceptible to peroxidation, and at the cellular level, by increasing storage in LDs. Thus, manipulating LD dynamics represents a promising strategy to overcome hypoxia-induced resistance to ferroptosis and improve the success of PCa treatment.

## INTRODUCTION

Ferroptosis is a form of cell death that involves lipid peroxidation mediated by iron, and dysregulation of ferroptosis is implicated in numerous human pathologies, including cancer [1–3]. Ferroptosis is regulated by a complex transcriptional and post-translational signaling network that balances iron homeostasis, lipid metabolism, and the cellular redox system. Dysregulation of these processes can result in the accumulation of toxic phospholipid (PL) peroxides in the cell membrane due to oxidation of PLs containing polyunsaturated fatty acids (PL-PUFAs). The primary antioxidant system protecting against ferroptosis is the Xc^–^-GSH-GPX4 pathway. As a result, pharmaceutics targeting this system are among the most successful ferroptosis inducers (FINs) developed to date. Two major categories of redox-mediated FINs are cysteine/glutamate (xCT) antiporter inhibitors (i.e., Erastin) and direct inhibitors of glutathione peroxidase 4 (GPX4). Blocking the xCT system induces ferroptosis by reducing GSH levels and increasing reactive oxygen species (ROS) [3–7]. Erastin has been shown to increase the sensitivity of various solid tumor cell lines to chemotherapies [8–11]. GPX4 is the central antioxidant enzyme responsible for suppressing ferroptosis by transforming lipid ROS into lipid alcohols. As a result, direct GPX4 inhibitors (RSL3) have been developed that drive ferroptosis independent of intracellular cysteine and GSH levels [12]. However, as the use of FINs has expanded, new mechanisms of resistance are emerging [13]. Therefore, understanding how tumor cells adapt and escape ferroptosis is necessary to maximize the effect FINs as an anti-cancer therapeutic strategy [14].

All solid tumors experience hypoxia, which leads to a plethora of transcriptional and post-translational changes that allow for survival [15, 16]. This is particularly true of prostate cancer (PCa), which arises in the hypoxic peripheral zone. The hypoxic nature of the prostate has been demonstrated by spatial phenotyping of hypoxic gene expression signatures [17] and non-invasive imaging methods (i.e., PET/MRI) [18]. The peripheral zone of the prostate becomes hypoxic due to decreased blood flow, which has been reported using non-invasive multi-parametric MRI procedures and with newer blood oxygen level-dependent imaging (BOLD MRI). Notably, clinical studies have established that the presence of hypoxia biomarkers and gene signatures in the primary tumor is associated with worse overall and progression free survival in PCa patients [19, 20]. While hypoxia has been implicated in resistance to ferroptosis in cancer, the mechanisms vary [21] and effect of hypoxia on lipid metabolism in relation to ferroptosis resistance in PCa remains unclear.

Clinical studies demonstrate that metabolic reprogramming of fatty acids, phospholipids, and cholesterol are critical for PCa progression and metastasis [22]. The accumulation of cholesteryl esters (CEs) correlates with resistance to androgen deprivation therapy (ADT) and increased metastasis [23] and targeting cholesterol esterification suppressed PCa metastasis [24, 25]. The storage of such neutral lipids is mediated by lipid droplets (LDs), dynamic organelles that structurally consist of a neutral lipid core (triglycerides and CEs) surrounded by a phospholipid monolayer. The link between LDs and ferroptosis is emerging, but the role of hypoxia and LDs in mediating sensitivity to FINs in PCa has not been investigated.

Here, we profile the transcriptional and cellular changes associated with hypoxia and ferroptosis resistance in PCa. Our results show that hypoxia significantly reduces sensitivity to FINs by altering the expression of metabolic genes to favor the production of lipids that are less susceptible to peroxidation, while simultaneously enhancing LDs to sequester PUFAs, reduce membrane lipid peroxidation, and prevent ferroptosis. Thus, manipulating LD dynamics could be a promising strategy to increase the efficacy of FINs as anticancer therapies, alone and in combination with chemotherapy.

## RESULTS

### Hypoxia promotes resistance to ferroptosis inducing agents in prostate cancer

**Figure 1:**
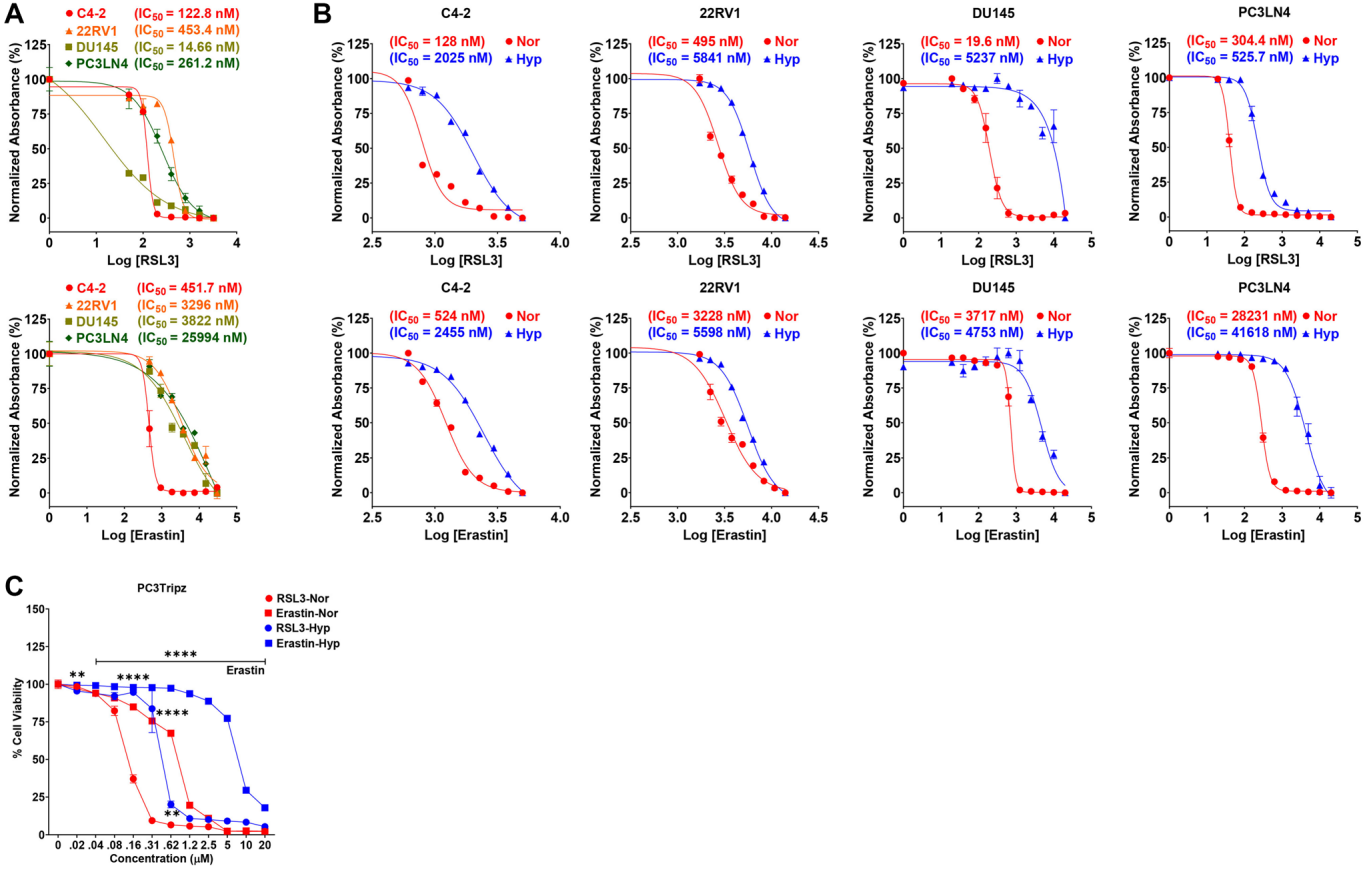
Hypoxia promotes resistance to ferroptosis in prostate cancer. (**A**) IC_50_ graphs for ferroptosis inducers (FINs) in a cell line panel from prostate cancer (PCa; RSL3 (0–3.2 μM); Erastin (0–30 μM)). (**B**) IC_50_ graphs for FINs in PCa panel under normoxia (Nor) or hypoxia (Hyp). (**C**) Dose response curve for FINs in PCa cells under Nor or Hyp. (A–C) Cells were treated for 72 h as indicated and processed for CV staining to calculate cell viability and IC_50_ values or dose response curves using Prism GraphPad. *n* = 3, mean ± standard deviation (SD) ^****^ *P* < 0.0001, ^***^ *P* < 0.001, ^**^ *P* < 0.01, and ^*^ *P* < 0.05 by One-way ANOVA or Student’s *t*-test.

To determine how hypoxia impacts the efficacy of FINs, a panel of PCa cell lines were maintained in normoxia (Nor, 20% O_2_) or hypoxia (Hyp, 1.0% O_2_) and treated with increasing concentrations of FINs. PCa cells lines with common genetic alteration (C4-2, 22RV1, DU145, and PC3LN4) were chosen to represent the spectrum of the disease, as well as a normal prostate epithelial line (RWPE1). Each cell line was treated with increasing concentrations of RSL3 or Erastin in normoxia or hypoxia, and viability was measured using crystal violet (CV). As expected, the PCa cell lines showed differential sensitivity to RLS3 and Erastin under basal conditions (Figure 1A). The IC_50_ values for FINs were significantly higher in hypoxic cells (Figure 1B) providing evidence that hypoxia suppresses ferroptosis. This is also reflected by the response to FINs at higher dose under hypoxia (Figure 1C). Interestingly, the RSL3 IC_50_ was significantly higher in RWPE1 (Supplementary Figure 1A) compared to all PCa cell lines. To determine if the observed effects are specific to prostate cells or generally applicable to other solid tumors, lung cancer cell lines (H1299, H1993, H460 and A549) and a normal lung epithelial line (BEAS2B) were treated with FINs, as described previously. Like PCa cells, lung cell line models showed differential sensitivity to FINs (Supplementary Figure 1B, 1C) and exhibited dose response at higher concentrations of RSL3 or Erastin (Supplementary Figure 1D). However, BEAS2B showed similar or higher sensitivity to FINs compared to cancer cells (Supplementary Figure 1B, 1C), further highlighting the context-dependent nature of ferroptosis [26]. These data demonstrate that hypoxia is a significant cause of resistance to FINs in normal and solid tumor (prostate/lung) cell lines, regardless of their basal sensitivity.

### Co-targeting GPX4 and xCT has synergistic cytotoxic effects that are blocked in hypoxia

**Figure 2:**
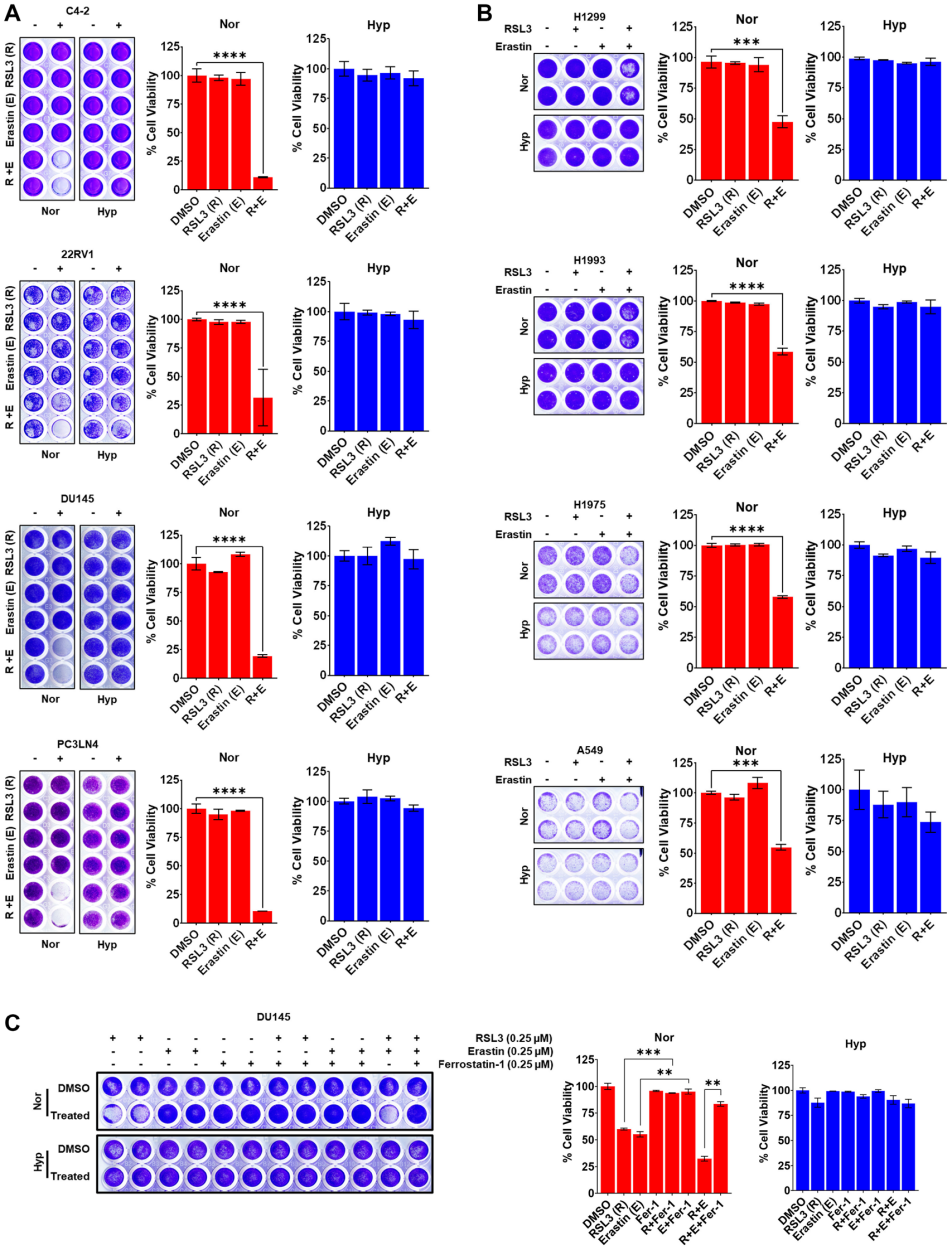
Co-targeting GPX4 and xCT has synergistic cytotoxic effects that are blocked in hypoxia. (**A**–**C**) Representative images of crystal violet (CV) staining in cells as indicated under normoxia (Nor) or hypoxia (Hyp) treated with ferroptosis inducers (FINs), RSL3 or Erastin; or inhibitor (Ferrostatin-1) alone, or together. Histograms represent relative cell viability under indicated treatment conditions quantified from CV staining. (A–C) Cells were treated for 72 h and processed for CV staining to calculate cell viability using Prism GraphPad *n* = 3, mean ± standard deviation (SD) ^****^ *P* < 0.0001, ^***^ *P* < 0.001 by or Student’s *t*-test.

Hypoxia reduced sensitivity to both RSL3 and Erastin, even though they induce ferroptosis through different mechanisms. Thus, we tested if simultaneously targeting GPX4 and glutathione depletion could further sensitize PCa to ferroptosis and/or overcome hypoxia-mediated resistance. To this end, normal and cancerous prostate and lung cell lines were conditioned in normoxia or hypoxia and treated with sub-toxic dose of RSL3 (0.1 μM) or/and Erastin (0.1 μM). In normoxia, neither FINs reduced viability as a monotherapy, but the combination produced nearly complete cell killing. However, synergy between RSL3 and Erastin was abrogated under hypoxia in all cell line tested (Figure 2A, 2B). Finally, to confirm that the cytotoxicity of FINs is due to ferroptosis, DU145 PCa cells were treated with FINs in the presence or absence of Ferrostatin-1 (Fer-1), a pharmacological inhibitor of ferroptosis, and cell viability was measured. As expected, FINs were more effective in normoxia than hypoxia and combination treatment with Erastin and RSL3 was synergistic (Figure 2C). Treatment with Fer-1 significantly reduced the cytotoxicity of FINs in normoxia, demonstrating that ferroptosis is the primary pathway responsible for cell death (Figure 2C). In hypoxia, DU145 cells were significantly less sensitive to ferroptosis and required higher concentration of FINs to reduce cell viability. At higher doses, where co-treatment was effective in hypoxia, cell death was also rescued by Fer-1 (Supplementary Figure 1E; Supplementary File 1). Together, these results demonstrate that hypoxia is a powerful driver of resistance to FINs in solid tumors (prostate/lung).

### Hypoxia alters gene expression to favor sensitivity to ferroptosis

**Figure 3:**
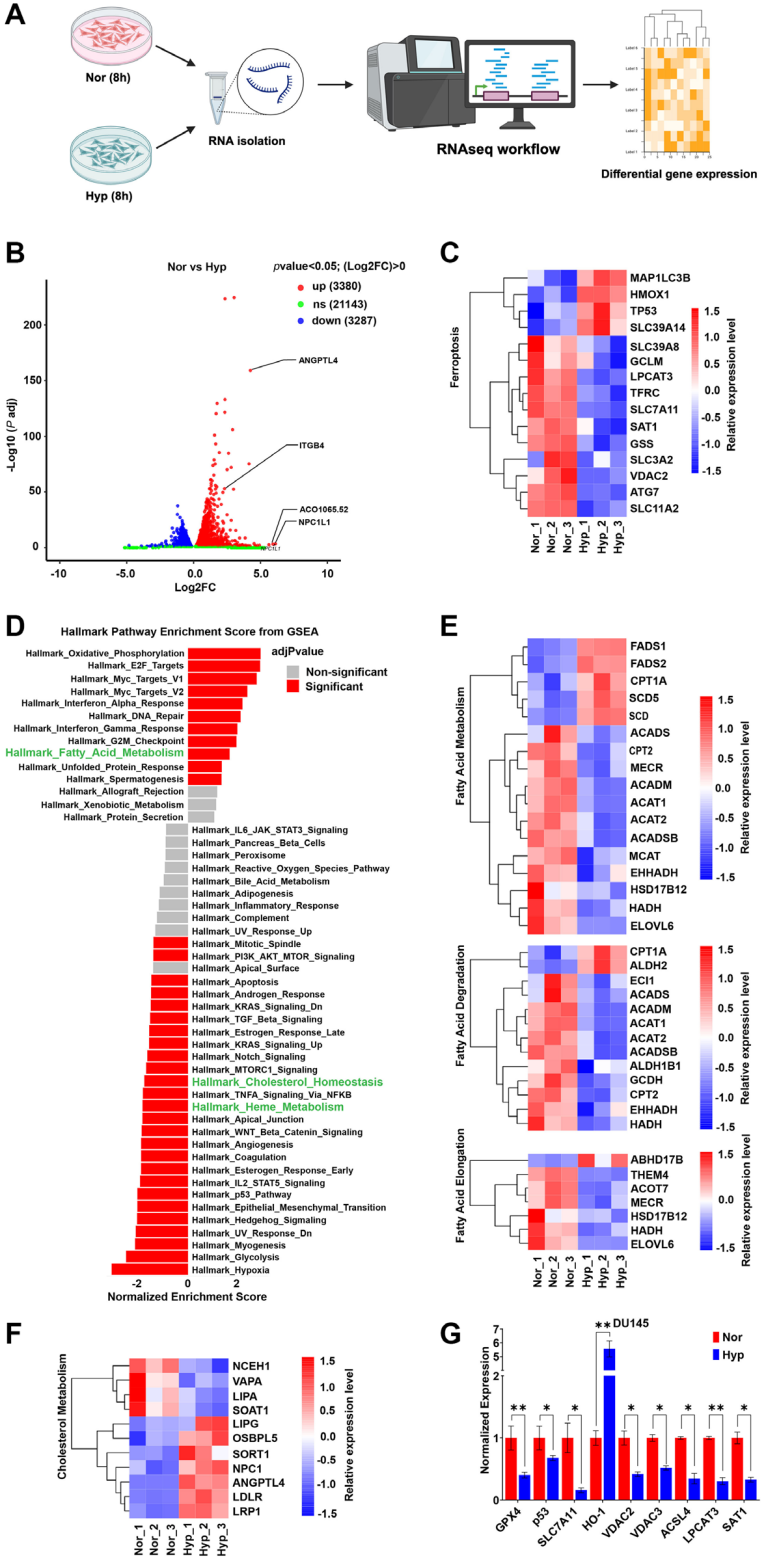
Hypoxia alters gene expression to favor sensitivity to ferroptosis. (**A**) representative schematics of sample preparation and workflow for RNA sequence analysis. (**B**) Volcano plot display differentially expressed genes (DEGs). Red (upregulated) and blue (downregulated) dots represent significant DEGs at indicated cutoffs (log2FC >4 and *p*-value < 0.05). (C, E, and F) Heat maps display significant DEGs relating to ferroptosis pathway (C), fatty acid metabolism (E), and cholesterol metabolism (F) at cutoffs (log2FC >4 and *p*-value < 0.05). (D) GSEA enrichment score plot showing significant (Red) gene sets. (**B**–**F**) PC3 cells were maintained in normoxia (Nor) or hypoxia (Hyp) for 8 h and analysis performed Nor vs. Hyp, *n* = 3. (**G**) Relative normalized expression of indicated genes relating to GPX4 regulation (GPX4, p53, and SLC7A11), iron metabolism (HO-1, VDAC2, and VDAC3), and lipid metabolism (ACSL4, LPCAT3, and SAT1) in DU145 cells under hypoxia for 8 h. *n* = 3, mean ± standard deviation (SD) ^**^ *P* < 0.01, and ^*^ *P* < 0.05 by Student’s *t*-test.

Published studies in cancer and other cell types have shown that hypoxia-induced gene expression (primarily downstream of hypoxia inducible factors 1 and 2 (HIF-1 and HIF-2)) can alter genes to promote or suppress ferroptosis. To determine how hypoxia is impacting ferroptosis-related genes in PCa cells, PC3 were cultured in normoxia or hypoxia for 8 h, RNA was collected for transcriptomic analysis (Figure 3A). RNA-seq analysis revealed a total of 27,810 DEGs (3,380 up; 3,287 down; and 21,143 unaltered) with log2FoldChange (log2FC) >0 and *p*-value < 0.05 (Supplementary Tables 1 and 2; Supplementary File 2). To isolate the most significant expression changes, we applied a more stringent cut-off (log2FC >4 and *p*-value < 0.05), and volcano plots were generated to highlight potential hits (Figure 3B). Next, we assessed the effect of hypoxia on a curated panel of 15 genes that are involved during ferroptosis. As expected, hypoxia reduced the expression of most ferroptosis-related genes (SLC39A8, GCLM, LPCAT3, TFRC, SLC7A11, SAT1, GSS, SLC3A2, VDAC2, ATG7, and SLC11A2), with some exceptions (MAP1LC3B, HMOX1, TP53, SLC39A14) (Figure 3C). Gene Set Enrichment Analysis (GSEA) revealed that genes involved in fatty acid metabolism and cholesterol homeostasis were among the most prevalent alterations (Figure 3D). Fatty acid metabolism is essential for ferroptosis, as it is responsible for the synthesis of PUFAs, [27, 28], which is the primary substrate peroxidation [29]. Surprisingly, hypoxia altered genes related to fatty acid degradation and elongation. (Figure 3E) [30] as well as genes in cholesterol metabolism which could be implicated in ferroptosis (Figure 3F). Hits from RNA-seq analysis were validated by qPCR, and we confirmed that hypoxia reduced the expression of genes that inhibit lipid peroxidation (GPX4, p53, and SLC7A11), iron metabolism (VDAC2, and VDAC3), and lipid metabolism (ACSL4, LPCAT3, and SAT1) (Figure 3G). We also noticed that hypoxia per se does not affect GPX4 at protein level (Supplementary Figure 1F; Supplementary File 1), but RSL3 significantly reduces protein expression (Supplementary Figure 1G; Supplementary File 1). Paradoxically, these results suggest that global changes in hypoxic PCa cells would favor enhanced susceptibility to ferroptosis, not resistance, suggesting that the relevant changes are likely occurring at the lipid level.

### Hypoxia reduces the levels of neutral lipids available for peroxidation

**Figure 4:**
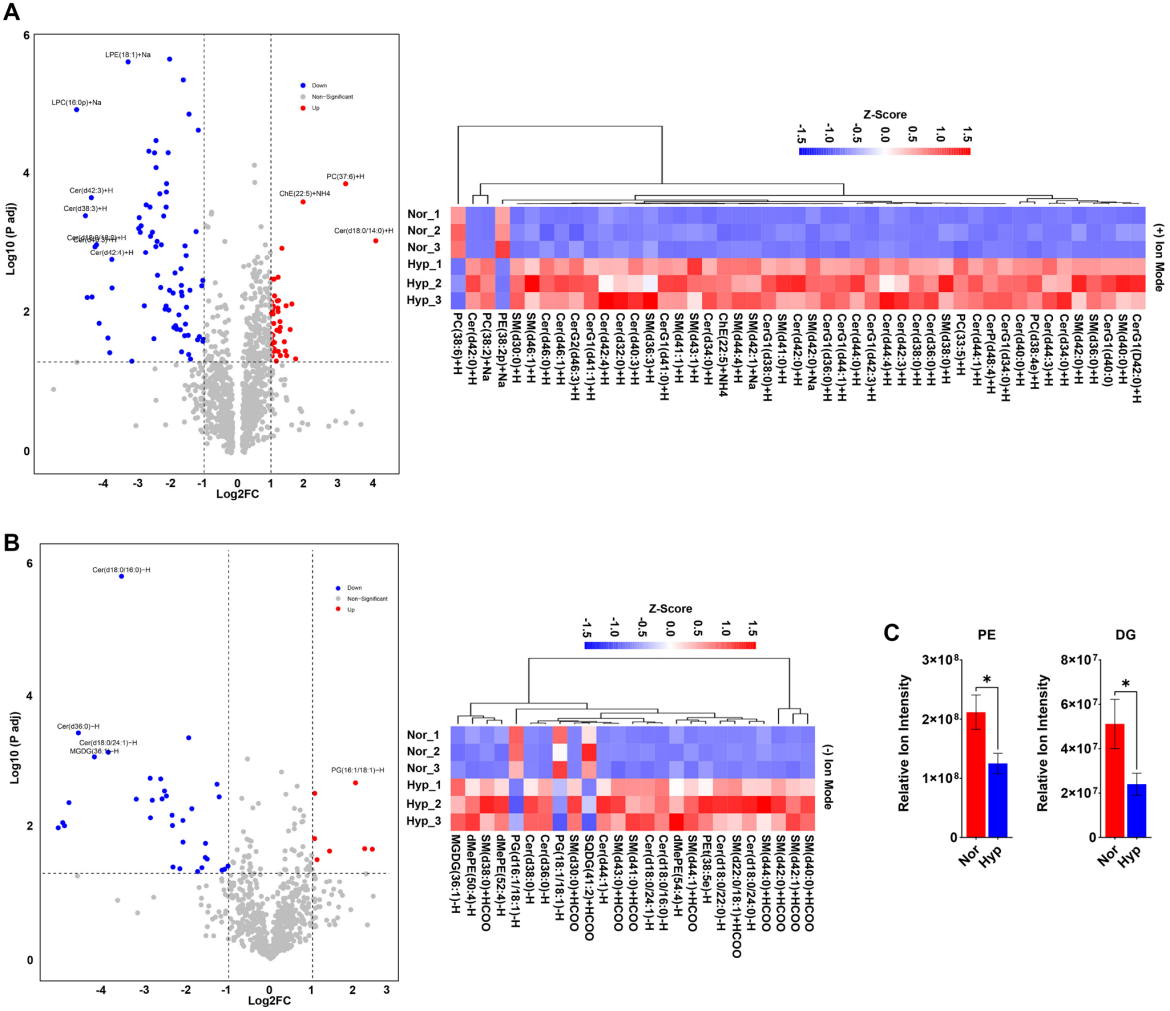
Hypoxia reduces the levels of neutral lipids available for peroxidation. (**A**, **B**) Volcano plot display significantly altered (Y>1.30, X>1, increase, red dots and Y>1.30, X<-1, decrease, blue dots) lipid species from positive (A) or negative (B) ion mode non-targeted lipidomic analysis. Heat maps represent hierarchical cluster analysis of significant lipids with VIP score >1.5 from non-targeted lipidomic analysis as indicated. PC3 cells were maintained in normoxia (Nor) or hypoxia (Hyp) for 8 h and analysis performed Nor vs. Hyp, *n* = 3. (**C**) Histograms showing significantly altered lipid classes (PE and DG) in PC3 cells in hypoxia for 8 h. *n* = 3, mean ± standard deviation (SD) ^*^ *P* < 0.05 by Student’s *t*-test.

Altered lipid metabolism is common in cancer [31, 32] and can dictate the sensitivity of cells to ferroptosis induction [33]. Our transcriptomic data identify that downregulation of fatty acid metabolism and cholesterol homeostasis as potential nodes by which hypoxia could influence the lipid profile to alter sensitivity to FINs. To determine how hypoxia changes lipid composition, PC3 cells were conditioned in hypoxia for 8 h and samples were collected for untargeted lipidomic analysis. The resulting data were analyzed using partial least squares-discriminate analysis (PLS-DA) to identify changes in the relative amounts of lipids. There was a pronounced separation between normoxic and hypoxic samples by PLS-DA score plot (Supplementary Figure 2A, 2B; Supplementary File 1), indicating a significant change in lipid species. A total of 1,818 lipid species were identified (1,053, positive ion mode, (Supplementary Table 3A; Supplementary File 2); 765, negative ion mode, (Supplementary Table 4A; Supplementary File 2)). The volcano plot highlights the most significantly upregulated (PC(37:6)+H, ChE(22:5), Cer(d18:0/14:0)+H, and PG(16:1/18:1)-H) and downregulated (LPE(18:1)+Na, LPC(16:0p)+Na, Cer(d42:3)+H, Cer(d38:3)+H, Cer(d42:4)+H, Cer(d18:0/16:0-H, Cer(d36:0)-H, Cer(d18:0/24:1), and MGDG(36:1)-H) lipids under hypoxia (Figure 4A, 4B). Heat maps showing lipids with VIP score >1.5 from positive and negative ion modes were generated using hierarchical cluster analysis (HCA) (HCA) (Supplementary Tables 3B and 4B; Supplementary File 2). To examine the relationship between hypoxia and the identified lipids, a correlation network diagram was generated using the KEGG database. All significant lipids were imported, annotated, and pathway enrichment analysis was performed. The data revealed enrichment of dihydroceramides (DHCer), sphingomyelins (SM), Glycerophosphoglycerols, phosphosphingolipids, and Glycerophosphocholines in hypoxic cells (Supplementary Figure 2C, 2D; Supplementary File 1; Supplementary Tables 3C and 4C; Supplementary File 2). Regarding ferroptosis, hypoxia significantly decreased lipid classes containing PUFAs, most notably PE and PC species (Figure 4C, Supplementary Figure 2E, 2F; Supplementary File 1), which are susceptible to peroxidation and have been reported to enhance ferroptosis when present in excess [34]. These results suggest that hypoxia imparts resistance to FINs by reducing the levels of key PUFAs that drive ferroptosis.

### Hypoxia induces LD accumulation to promote ferroptosis resistance

**Figure 5:**
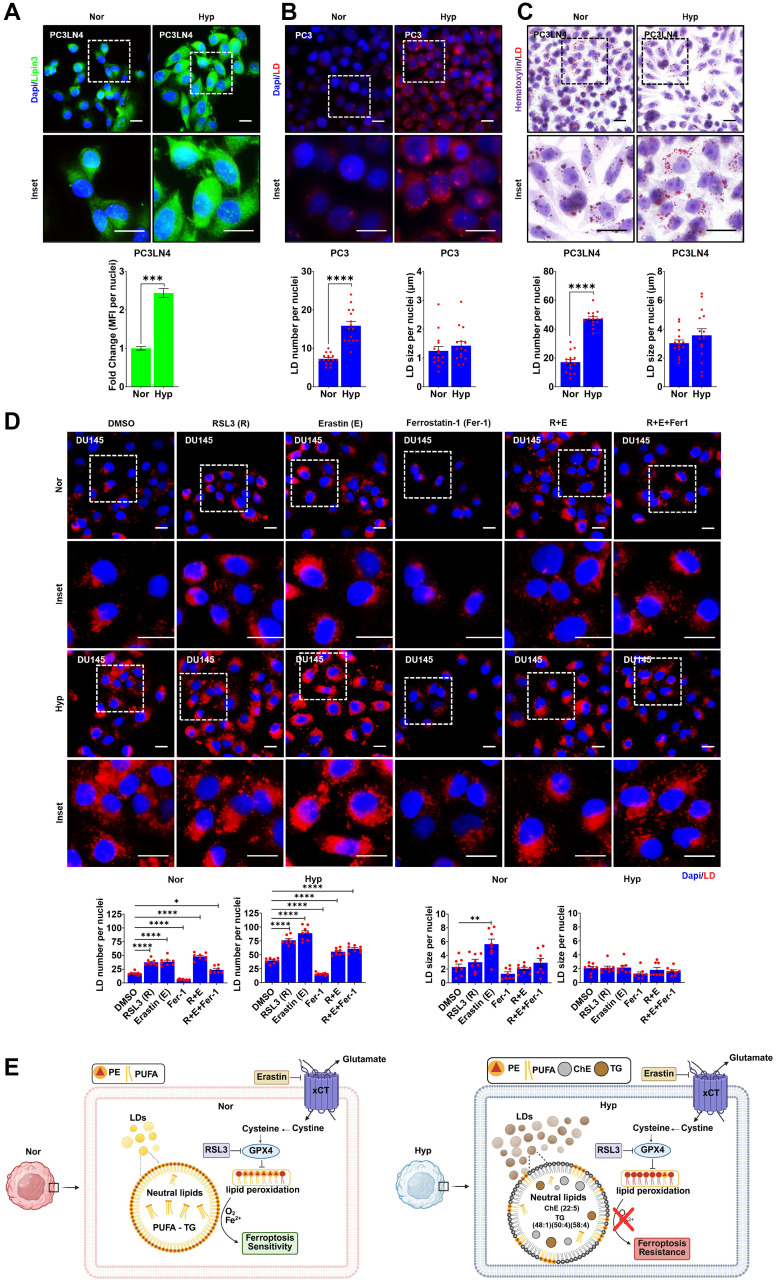
Hypoxia induces lipid droplets (LD) accumulation to promote ferroptosis resistance. (**A**) Representative images of Lipin-3 immunofluorescence analysis in PC3LN4 cells. Scale bars, 20 μm, magnification 60X. (**B**–**D**) Representative images of LDs in cells with treatments as indicated. LD stain, LipidSpot610 ((B) and (D)) and Oil Red O (C). *n* = 3, scale bars, 20μm, magnification 60X. (**E**) Working model describing the role of hypoxia in promoting ferroptosis resistance through LDs. (A–D) Histograms represent quantification of immunofluorescence (A) and LD number or size per nuclei (B–D). Indicated cells were maintained in normoxia (Nor) or hypoxia (Hyp) for 8 h (A), (B), and (D)) or 2 h (C). DU145 cells were treated with 1 μM of RSL3 (R), Erastin (E), Ferrostatin-1 (Fer-1), R+E, and R+E+Fer-1 (D). *n* = 3, mean ± standard deviation (SD) ^*^ *P* < 0.05, ^**^ *P* < 0.01, ^***^ *P* < 0.001, and ^****^ *P* < 0.0001 by Student’s *t*-test.

Accessibility to PUFAs is a rate limiting step for ferroptosis. When PUFAs are in excess, cells attempt to sequester them into lipid droplets (LDs) to prevent unwanted lipid peroxidation. Based on the increase in neutral lipids observed in hypoxic cells in our lipidomic analysis (Supplementary Figure 2G, 2H; Supplementary File 1), we hypothesized that hypoxia could sequester certain neutral lipids in LD, which would reduce their availability for lipid peroxidation and subsequently promote resistance to FINs. To test whether hypoxia induces LDs in PCa, PC3 and PC3LN4 cells were cultured in hypoxia for 8 h prior staining for lipin-3, a lipase that breaks down lipids in LDs and release fatty acids, and a dye that binds to LDs (LipidSpot610). Strikingly, hypoxia elevated the expression of Lipin3 (Figure 5A), which correlated with enhanced LD accumulation (Figure 5B, 5C). To determine the kinetics of LD accumulation in hypoxia, the indicated cells were grown in hypoxia for 2 h (acute hypoxia) or 5, 12, and 26 days (chronic hypoxia) prior to staining with Oil Red O to measure LDs. Within 2 h of hypoxia, the number of LDs significantly increased, and this trend continued over time in hypoxia (Figure 5C and Supplementary Figure 3A; Supplementary File 1). Interestingly, PCa cells in chronic hypoxia displayed significantly larger LDs compared to acute (2 h) or extended hypoxia (5–12 days), suggesting that LD size and number may be important for modulating susceptibility to ferroptosis. To determine if there is a correlation between LDs and sensitivity to FINs, normoxic and hypoxic DU145 cells were treated with RSL3, Erastin, and Ferrostatin-1, alone and in combination for 8 h. Quantification of LD staining demonstrated that treatment with FINs increased the number of LDs in normoxia and, to a significantly greater extent, in hypoxia (Figure 5D). Notably, LD number was suppressed by the ferroptosis inhibitor (Fer-1) (Figure 5D). To determine if LDs are essential for the hypoxia-mediated resistance to ferroptosis, we treated cells with an inhibitor of DGAT1, an enzyme that is required for LD biogenesis, in combination with FINs. Surprisingly, targeting LD biogenesis did not sensitize DU145 PCa cells to FINs in hypoxia (Supplementary Figure 3B; Supplementary File 1). However, targeting LD biogenesis and de novo lipogenesis altered sensitivity to RSL3 in cell line dependent manner under hypoxia (Supplementary Figure 3C, 3D; Supplementary File 1) suggesting that the accumulation of LDs in hypoxia is due to impaired LD degradation. Overall, these data show that increased LD in hypoxia is correlated with resistance to FINs.

## DISCUSSION

Growing evidence indicates that ferroptosis plays an important role in suppressing solid tumor growth and progression [4, 35], and LD accumulation is known to favor survival in response to stresses in the TME, such as hypoxia [36, 37]. Thus, there is a significant crosstalk between LDs and ferroptosis in solid tumors, and understanding the impact of hypoxia on these processes is necessary to design more effective treatment strategies. Here, we identify hypoxia as a driver of ferroptosis resistance in a panel of normal and cancerous PCa and lung cell lines. As noted in other solid tumors, hypoxia reduced the expression of genes that inhibit lipid peroxidation iron metabolism and cholesterol metabolism, which are expected to impair ferroptosis. Interestingly, hypoxia caused a paradoxical repression of genes involved in fatty acid synthesis and elongation, which ultimately dictates the amount of PUFAs available for peroxidation. It has been established that the presence of PUFAs in phospholipids is required for ferroptosis based on the requirement of lipid remodeling enzymes that catalyze the insertion of fatty acids into membrane phospholipids. Based on the literature, this conflicting effect of hypoxia on ferroptosis genes is not unexpected. Most adaptive responses under hypoxic microenvironments are direct implications of hypoxia-inducible factor (HIF) signaling [15], but there are conflicting reports whether HIF-1 positively or negatively impacts ferroptosis based on cell type and context. For example, it was reported that HIF-1α, but not HIF-2α, is the primary driver of ferroptosis resistance under hypoxia in a variety of solid tumors [35]. Subsequent reports showed strong evidence that ferroptosis induction is HIF-2α dependent on colorectal cancer [38] clear-cell carcinomas [39]. Together, these findings support a model where transcriptional changes are not sufficient to explain hypoxia-induced resistance to ferroptosis, and post-translational signaling at the protein and lipid level are necessary.

One of the most successful strategies for ferroptosis induction has been the use of small molecule FINs, such as Erastin and RSL3 [3–7]. However, to our knowledge, simultaneously targeting GPX4 and xCT has not been reported. Here, we show that co-targeting GPX4 and xCT has striking, synergistic cytotoxic effects in a wide variety of cell line models. However, hypoxia abrogated the cytotoxic effects of co-targeting RSL3 and Erastin tested, suggesting that the adaptive reprogramming that occurs in hypoxia is likely hindering ferroptosis at multiple signaling axes. Metabolic reprogramming of lipids is crucial for prostate cancer progression [40]. Our results show that hypoxia ensue lipidomic alterations favoring ferroptosis resistance. Since hypoxia-associated changes relating to ferroptosis resistance in prostate cancer are less studied, we performed non-targeted lipidomic analysis to identify changes in lipid classes or species during hypoxia corelating to ferroptosis. Notably, the levels of phosphatidylethanolamine (PE) class and PE or phosphatidylcholine (PC) species decreased in PC3 PCa cells under hypoxia. Interestingly, lipid availability is known to influence ferroptosis sensitivity in cancer cells [34] and PUFA-rich PE or PC species are most susceptible to undergoing ferroptosis. PUFA are liberated from triglycerides (TGs) and converted to highly unsaturated PUFAs which then accumulate in membrane phospholipids including PE or PC to promote ferroptosis susceptibility. Moreover, LDs can further restrict availability or distribution of pro-ferroptosis lipid species. It is possible that the increase in cholesteryl esters under hypoxia leads to LD accumulation along with depleted pool of PUFA-rich PE or PC species essentially favoring ferroptosis resistance.

Aside from the direct changes to lipid species observed in hypoxia, we also demonstrated that hypoxia dramatically increases the number and size of LDs. Notably, this effect was rapid, with significant increases in LD numbers within 2 h, and this effect was further potentiated over time, with very large LDs observed after chronic hypoxia (26 days). Interestingly, FINs (RSL3 or Erastin) significantly increased LD accumulation in PCa cells in normoxia, and to a greater extent in hypoxia. To our knowledge, this is the first evidence that tumor cells enhance LDs in response to treatment with FINs, which dampen their efficacy. Notably, treatment with a ferroptosis inhibitor (Fer-1) suppressed the increase in LDs after FIN treatment in both normoxia or hypoxia. Taken together, these results suggest that a negative feedback loop exists where the association between LDs and sensitivity to FINs warrants further study, beta negative feedback loop where tumor cells enhance LDs in response to treatment with FINs. Thus, altering LD dynamics in combination with FINs may be needed to improve their effectiveness, particularly in hypoxic tumors. Targeting LD biogenesis using DGAT1 inhibitor has been proposed in combination with chemotherapy to increase cell death in prostate cancer [41] or to promote ferroptosis during tumor acidosis [42]. However, DGAT1 inhibitor had no effects on the sensitivity to FINs highlighting the context dependent nature of ferroptosis. It is possible that the LD accumulation in prostate cancer cells experiencing hypoxia is not because of biogenesis but decreased or suppressed degradation. This further implies that investigating LD dynamics could give us better insights into their role in ferroptosis during hypoxia.

Overall, our findings demonstrate that hypoxia drives ferroptosis resistance though alterations in lipid metabolism and distribution executed by LDs. We speculate that the suppression of LD degradation contributes to hypoxia-associated resistance to ferroptosis via affecting PUFA-rich PE or PC species availability (Figure 5E). Thus, further studies are warranted to block LD accumulation to sensitize prostate cancer and overcome ferroptosis resistance under hypoxia.

## MATERIALS AND METHODS

### Cell lines and culture conditions

All cell lines including prostate (C4-2, 22RV1, DU145, PC3LN4, PC3, PC3Tripz, and RWPE1) and lung (BEAS2B, H1299, H1993, H460, and A549) were cultured either in RPMI1640 or DMEM (Gibco, USA) supplemented with 10% Fetal Bovine Serum (catalogue number: A5670701, Gibco, USA) and 1% Penicillin-Streptomycin (catalogue number: 15070063, Gibco, USA) in a humidified atmosphere of 5% CO_2_/95% air at 37°C. For hypoxia experiments, cells were maintained in 1% O_2_ for desired time with or without indicated treatments. All cell lines were authenticated by short tandem repeat DNA profiling performed by the University of Arizona Genetics Core facility-Arizona Research Laboratories Division of Biotechnology at the University of Arizona. (http://uagc.arl.arizona.edu/). The cell lines were used for less than 50 passages, and they were routinely tested for mycoplasma contamination.

### Cell viability assay

The cell viability was measured by crystal violet staining to assess proliferation. Briefly, cells were plated in 96-well plates, treated with ferroptosis inducers, RSL3 (catalogue number: S8155, Selleckchem, USA) or Erastin (catalogue number: S7242, Selleckchem, USA); ferroptosis inhibitor, Ferrostatin-1 (catalogue number: S7243, Selleckchem, USA); LD biogenesis inhibitor, DGAT1 (catalogue number: PF-04620110, Cayman Chem, USA); de novo lipogenesis inhibitors including Xanthohumol (DGAT1 and DGAT2 inhibitor, catalogue number: S7889, Selleckchem, USA), TOFA (acetyl-CoA carboxylase α inhibitor, catalogue number: S6690, Selleckchem, USA), FAS inhibitor (catalogue number: S6666, Selleckchem, USA) as indicated for 72 h, fixed in 4% formaldehyde, and stained with 0.1% crystal violet (catalogue number: C581-25, Fisher Scientific, USA). The cells were then lysed in a 1% sodium dodecyl sulfate (catalogue number: BP166-500, Fisher Scientific, USA) solution, and absorbance was measured using a microplate reader at a wavelength of 595 nm.

### qRT-PCR analysis

Messenger RNA was isolated from cell lysates using the RNeasy Mini Kit (catalogue number: 74104, Qiagen, USA), and cDNA synthesized from each sample using the RT^2^ first strand synthesis kit (catalogue number: 3301401, Qiagen, USA). Changes in gene expression in response to hypoxia with or without indicated treatments were measured as follows: qRT-PCR reactions were performed with equal amounts of starting material (1000 ng RNA) using qPCRBIO SyGreen Blue Mix (PCR Biosystems, PB20.15-01), according to the manufacturer’s protocol. Validated human primer sets (OriGene Technologies) for each of the following genes were purchased to measure gene expression: GPX4, SLC3A2, SLC7A11, P53, SAT1, ALOX15B, ALOX15, LPCAT3, ACSL4, TFRC, SLC11A2, SLC39A8, HO-1, VDAC2, VDAC3, MAP1LC3B, NCOA4, and NOX1. All primers were ordered from OriGene and IDT (Supplementary Table 5; Supplementary File 2). GAPDH was used to normalize.

### Transcriptomics analysis

Transcriptomics analysis was performed on PC3 cells following 8 h incubation in normoxia or hypoxia. RNA was isolated as described above and checked for quality using Novogene’s QC method assessing purity, integrity, and quantity of RNA in each sample. Sequencing libraries were generated using Novogene’s standard mRNA-seq library preparation method involving a multistep (rRNA depletion, fragmentation, reverse transcription, ligation, and PCR amplification) process. Paired-end 150bp read strategy was employed for sequencing platform. Finally, raw reads were processed for gene expression quantification and differential gene expression analysis. An additional pdf file shows this in more detail.

### Non-targeted lipidomic analysis

Non-targeted lipidomic analysis was performed as described previously [43]. More details are included in the Materials and Methods section of Supplementary File 1.

### Immunofluorescence

Cells were plated in six-well plates containing microscope coverslips and treated as indicated. After treatment, cells were fixed with 4% formaldehyde for 20 min and kept in a blocking solution (5% NGS and 0.3% Triton X-100 in PBS) for 60 min. Then, cells were incubated with anti-Lipin 3 (catalogue number: PA5-48043, ThermoFisher Scientific, USA) antibody for 60 min. Following primary antibody incubation, cells were washed with 1X DPBS and incubated in secondary antibodies (Alexa Fluor 488 goat anti-mouse, 1:500 dilution) for 60 min. Finally, cells were mounted on glass slides with mounting media containing DAPI (catalogue number: 8961 S, Cell Signaling Technology, USA). For LD imaging, cells were seeded and fixed in 4% formaldehyde as described above followed by 30 min incubation in 1:1000 diluted LipidSpot610 (catalogue number: 70069, Biotium, USA). After staining, cells were washed in 1X DPBS and mounted onto slides. Images were taken at 60× magnification using a fluorescent microscope.

### Oil red O staining

Oil Red O staining was performed according to the manufacturer’s procedure (catalogue number: MAK194, Sigma-Aldrich, USA). Briefly, a stock oil red solution was prepared to dilute 0.7 g Oil Red O with 200 mL isopropanol. A working dilution was then obtained by mixing 6 parts Oil-Red O stock with 4 parts dH2O. Specimens (cells) were first fixed in 10% formalin for 30 min and incubated in 60% isopropanol for 5 min. Then, specimens were covered in a working oil red solution for 20 min and kept in hematoxylin for 1 min. At the end of each step, specimens were washed 2–5 times with dH2O. Finally, analyzed immediately by light microscopy.

### Western blotting

Cultured cells were lysed in RIPA buffer containing protease inhibitors. Protein concentration was determined by Bradford Protein Assay (catalogue number: 20830000-1, Bioworld, USA). Equal protein per well was loaded and resolved by SDS-PAGE. Gels were transferred onto PVDF membranes (catalogue number: 88518, Thermo Scientific, USA) and immunoblotted using the following antibodies: anti-GPX4 (catalogue number: 52455, CST, USA) and anti-GAPDH (catalogue number: 2118, CST, USA). Anti-rabbit (catalogue number: 7074, CST, USA) HRP secondary antibodies were used for detection. Blots were imaged on a ChemiDoc (SynGene) using chemiluminescence detection with ECL western blotting substrate (catalogue number: 34095, Thermo Scientific, USA).

### Statistical analysis

All data are presented as the mean ± standard error mean (SEM), and statistical analysis was performed using the GraphPad Prism version 10 software. Statistical significance was calculated using Student’s *t*-tests as well as by ANOVA unless stated otherwise in figure legends. Each experiment was conducted at least three times (*n* = 3). Values with *p*-value lower than 0.05 were considered statistically significant (^*^ *p* ≤ 0.05, ^**^ *p* ≤ 0.01, and ^***^ *p* ≤ 0.001).

## SUPPLEMENTARY MATERIALS





## Abbreviations

ACSL4: acyl-CoA Synthetase Long Chain Family Member 4
ALOX15: arachidonate 15-lipoxygenase
ALOX15B: arachidonate 15-lipoxygenase type B
ANGPTL4: angiopoietin-like 4
BOLD MRI: Blood oxygen level dependent Magnet Resonance Imaging
ChE: cholesteryl ester
CV: crystal violet
DGAT1: diacylglycerol o-acyltransferase 1
Fer-1: ferrostatin-1
FINs: ferroptosis inducers
GPX4: glutathione peroxidase 4
HO-1: heme oxygenase 1
Hyp: hypoxia
ITGB4: *integrin Subunit β 4*
LCa: lung cancer
LDs: lipid droplets
LPCAT3: *lysophosphatidylcholine acyltransferase 3*
MAP1LC3B: microtubule-associated protein 1 light chain 3β
NCOA4: *nuclear receptor coactivator 4*
Nor: *normoxia*
NOX1: *NADPH oxidase 1*
NPC1L1: Niemann-Pick C1-Like 1
P53: tumor protein 53
PC: phosphatidylcholine
PE: phosphatidylethanolamine
PCa: prostate cancer
PUFA: polyunsaturated fatty acids
SAT1: spermidine/spermine N(1)-acetyltransferase 1
SLC3A2: solute carrier family 3 member 2
SLC7A11: solute carrier family 7 member 11
SLC39A8: solute carrier family 39 member 8
SLC11A2: solute carrier family 11 member 2
TFRC: transferrin receptor protein
xCT: cysteine/glutamate antiporter system
VDAC2/3: voltage-dependent anion channel 2/3
