# Supplementary material for: Hypoxia induced lipid droplet accumulation promotes resistance to ferroptosis in prostate cancer
Source: Oncotarget. 2025 Jun 25;16:532–44. doi: 10.18632/oncotarget.28750 (PMC12196323; doi:10.18632/oncotarget.28750)
Supplement: Supplementary file 1 [file oncotarget-16-28750-s001.pdf]

Hypoxia induced lipid droplet accumulation promotes resistance to ferroptosis in prostate cancer

SUPPLEMENTARY MATERIALS

MATERIALS AND METHODS

Non-Targeted lipidomic analysis

Part 1: Sample preparation

Samples and groups

Total 6 human prostate cancer cell line (PC3) samples collected at the end of 8 h normoxia (Nor) or hypoxia (Hyp) treatment group (*n* = 3).

Sample pretreatment

(1) The samples were thawed on ice
(2) Add 0.5 mL water, 1.5 mL chloroform/methanol (2:1, v/v) into each sample
(3) Vortex for 1 min and then centrifuge at 3000 rpm 4°C for 10 min
(4) Transfer the organic layer into a new sample tube, dry under nitrogen gas
(5) Resuspended with 200 μL isopropanol/methanol (v/v = 1:1), then add 5 μL internal standard LPC (12:0) (125 μg/mL)
(6) Centrifuge at 12000 rpm 4°C for 10 min
(7) Collect the supernatant for LC-MS analysis

Part 2: Instrument parameters

LC Parameters

Samples were separated on a Thermo Ultimate 3000 LC system equipped with a Phenomenex Kinetex C18 column (100 mm × 2.1 mm, 1.7 μm). Solvent A was acetonitrile/water (v/v = 6:4) containing 10 mmol/L ammonium formate. Solvent B was acetonitrile/isopropanol (v/v = 1:9) containing 10 mmol/L ammonium formate. The flow rate was 0.3 mL/min and the column oven was held at 50°C. 2 μL of each sample was injected. The gradient conditions used are shown in the table below.

Time (min)	A (%)	B (%)
0	70	30
10.5	0	100
12.5	0	100
12.51	70	30
16	70	30

MS Parameters

Column eluent was introduced to a Thermo Q Exactive mass spectrometer. The mass spectrometer was operated in positive ion mode and negative ion mode respectively with full scan MS at 70,000 resolution and data-dependent MS/MS collected from 200–1200 m/z at 17,500 resolution. The electrospray ionization source was maintained at a spray voltage of 3kV at positive ion mode and −2.8kV at negative ion mode with sheath gas at 35 and auxillary gas at 15 (arbitrary units). The inlet of the mass spectromter was held at 350°C, and the S-lens was set to 50%. Samples were separated on a Thermo Ultimate 3000 LC system equipped with a Phenomenex.

Part 3: Analytical results

Multivariate statistical analysis

All the peaks in ESI+ were merged and imported into the SIMCA-P software for multivariate statistical analysis. To investigate the global lipids variations, we first use PCA to analyze all observations acquired in both ion modes. In order to eliminate any non-specific effects and confirm the biomarkers, PLS-DA or OPLS-DA to compare lipid changes in the two groups, respectively were performed. Finally, the significantly changed lipids (ions) between the treatment groups were filtered out based on VIP values (VIP >1.5).

Single variable analysis

We selected lipids with VIP >1.5, FC >1.0 and *p*-value < 0.05 as significant compounds for hierarchical cluster analysis. Univariate analysis including fold change analysis and *t*-test were performed on volcano plot.

Cluster analysis

Mean values of lipid contents from biological replicates in each treatment group were used to calculate lipid ratio. After log transformation of the data, median centered ratio was normalized. Hierarchical clustering analysis (HCA) was performed using the complete linkage

algorithm of the program Cluster 3.0 (Stanford University) and the results were visualized using Treeview (Stanford University). Lipids ratios from two independent experiments of all significant lipids were used for HCA. Color intensity correlates with degree of increase (red) and decrease (blue) relative to the mean lipid ratio. We selected significant lipids with VIP >1.5, FC >1.0 and p-value < 0.05 for hierarchical cluster analysis.

Pathway analysis

To investigate the latent relationships of the lipids, we constructed a correlation network diagram based on the KEGG databases. All significant lipids were imported to obtain categorical annotations, including pathways. We selected significant lipids with VIP >1.5, FC >1.0 and p-value < 0.05 for pathway analysis.

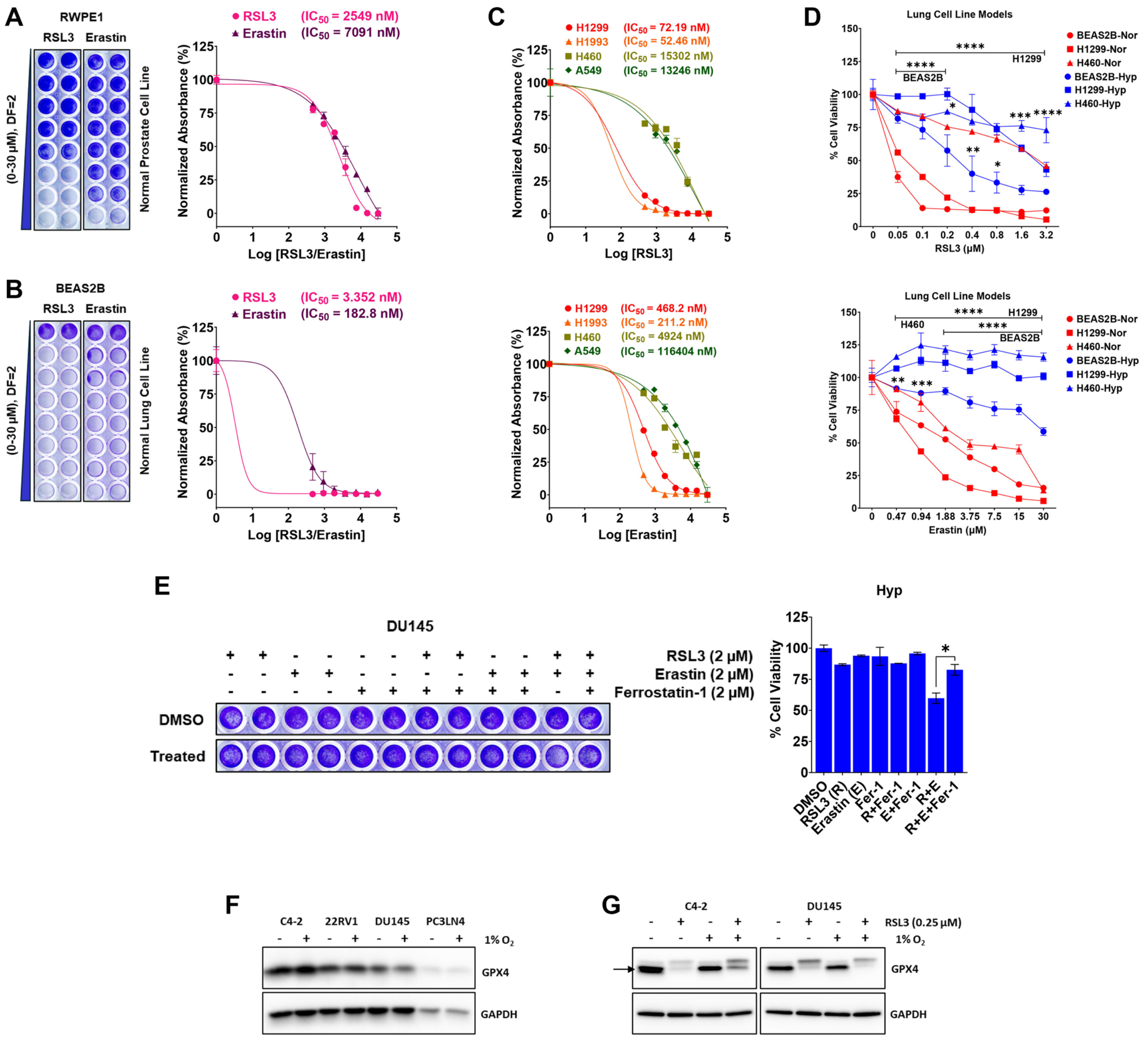

Supplementary Figure 1: Hypoxia promotes resistance to ferroptosis. (**A**, **B**) Representative images of crystal violet (CV) staining and IC_{50} graphs for ferroptosis inducers (FINs), RSL3 or Erastin in immortalized prostate (A) and lung (B) epithelial cells lines. Cells were treated for 72 h as indicated and processed for CV staining to calculate cell viability and IC_{50} values using Prism GraphPad. (**C**) IC_{50} graphs for FINs in a cell line panel from lung cancer (LCa; RSL3 (0–3.2 μM); Erastin (0–30 μM)). (**D**) Dose response curve for FINs in lung cell lines as indicated under Normoxia (Nor) or Hypoxia (Hyp). (**E**) Representative images and quantification of CV staining in DU145 cells treated as indicated under hypoxia for 72 h and cell viability was measured using Prism GraphPad. Western blot analysis for glutathione peroxidase 4 (GPX4) under Nor or Hyp alone (**F**) and with RSL3 (**G**). $n = 3$, mean ± standard deviation (SD), $^{*}P < 0.05$ by Student's t-test.

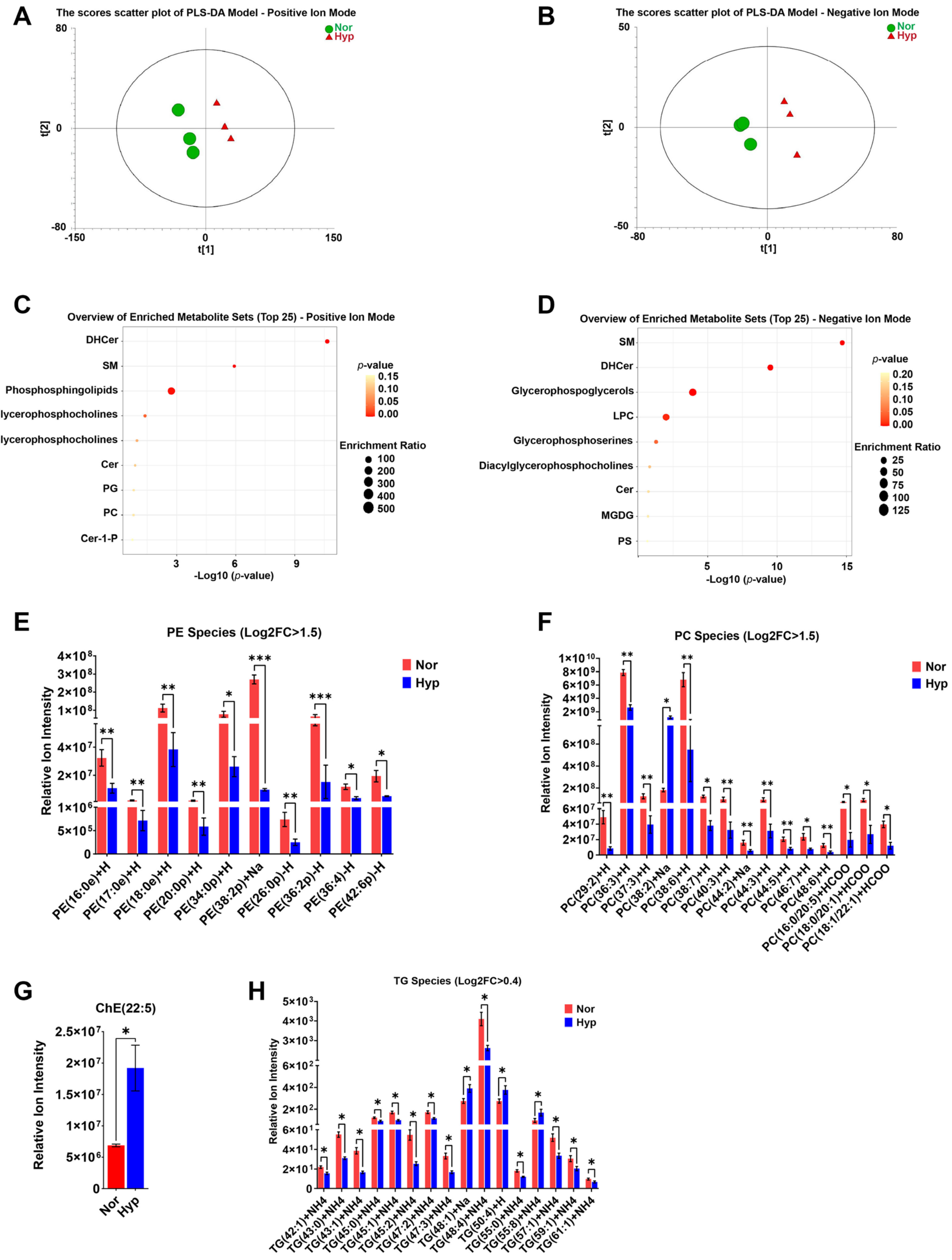

Supplementary Figure 2: Hypoxia reduces the levels of neutral lipids available for peroxidation. (**A**, **B**) The scores scatter plot of PLS-DA model from positive ion mode (A) or negative ion mode (B). non-targeted lipidomic analysis. (**C**, **D**) Overview of enriched metabolite sets from positive ion mode (C) or negative ion mode (D). non-targeted lipidomic analysis. (**E**–**H**) Histograms represent relative ion intensity of phosphatidylethanolamine (PE) (E), phosphatidylcholine (PC) (F), cholesteryl ester (ChE) (G), and Triglycerides (TG) (H). (A–H) PC3 cells were maintained in normoxia (Nor) or hypoxia (Hyp) for 8 h and non-targeted lipidomic analysis was performed using positive or negative ion mode. $n = 3$, mean ± standard deviation (SD), $^{*}P < 0.05$, $^{**}P < 0.01$, and $^{***}P < 0.001$ by Student's t-test.

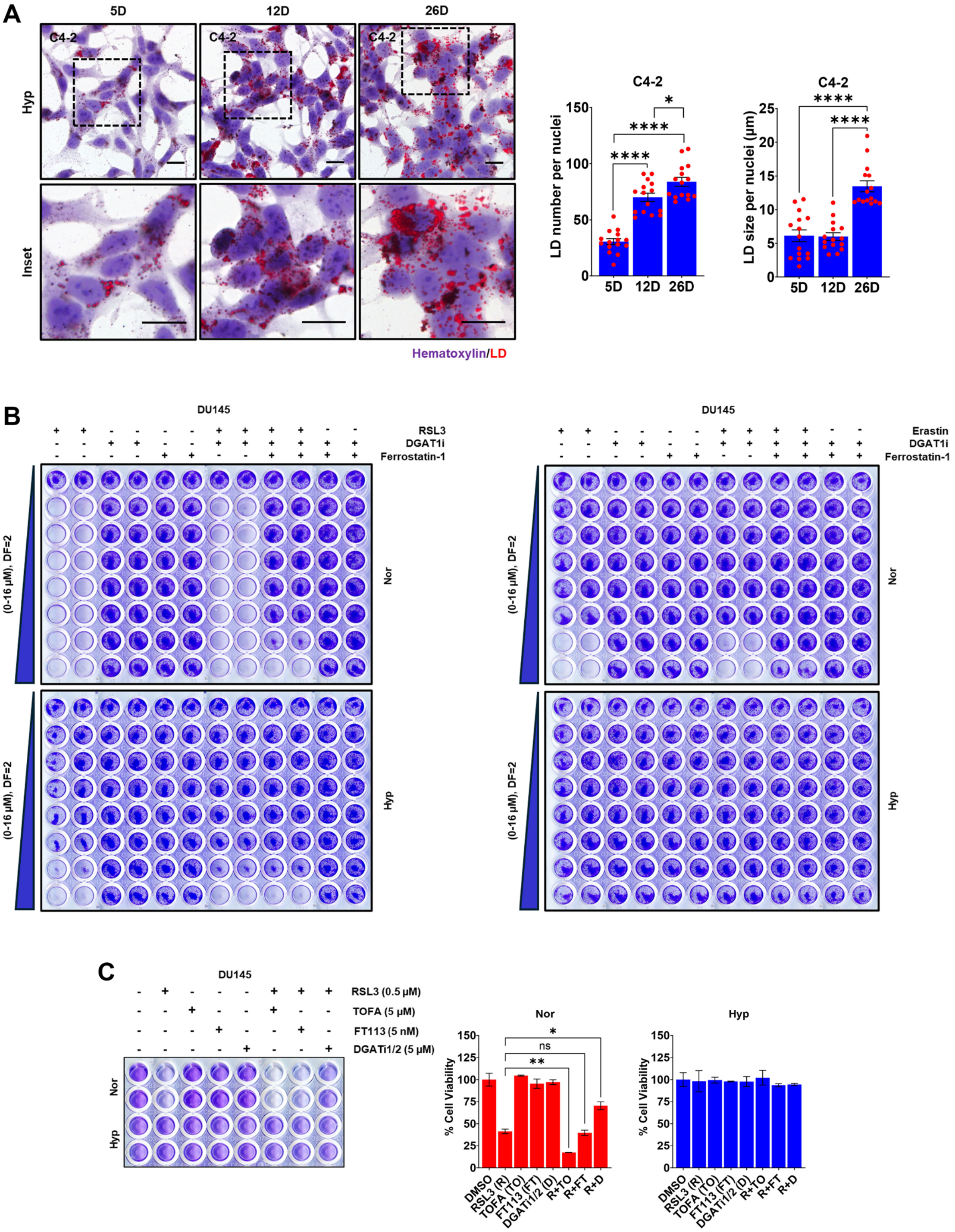

Supplementary Figure 3: Long term effects of chronic hypoxia on LD accumulation in prostate cancer. (**A**) Representative images of LDs in C4-2 cells following day (D) 2, 12, and 26 conditioning in hypoxia (Hyp). LDs are in red (Oil Red O) and nuclei in blue (Hematoxylin). Scale bars 50 µm, magnification 40X. $n = 3$, mean ± standard deviation (SD) $^{*}P < 0.05$, and $^{****}P < 0.0001$ by Student's *t*-test. (**B**, **C**) Representative images of crystal violet staining in prostate cancer cells treated as indicated under normoxia (Nor) or Hyp for 72 h. (C) Histograms represent quantification of CV staining. $n = 3$, mean ± standard deviation (SD) $^{*}P < 0.05$, and $^{**}P < 0.01$ by Student's *t*-test.

Supplementary Table 1: Upregulated differential gene expression under hypoxia in PC3 prostate cancer cells. See Supplementary Table 1.

Supplementary Table 2: Downregulated differential gene expression under hypoxia in PC3 prostate cancer cells. See Supplementary Table 2.

Supplementary Table 3A: Lipidomic alterations under hypoxia in PC3 prostate cancer cells (possitive ion mode non-targeted lipidomics). See Supplementary Table 3A.

Supplementary Table 3B: Hierarchical cluster analysis of significant lipids under hypoxia in PC3 prostate cancer cells (possitive ion mode non-targeted lipidomics). See Supplementary Table 3B.

Supplementary Table 3C: Pathway of enrichment analysis under hypoxia in PC3 prostate cancer cells (possitive ion mode non-targeted lipidomics). See Supplementary Table 3C.

Supplementary Table 4A: Lipidomic alterations under hypoxia in PC3 prostate cancer cells (negative ion mode non-targeted lipidomics). See Supplementary Table 4A.

Supplementary Table 4B: Hierarchical cluster analysis of significant lipids under hypoxia in PC3 prostate cancer cells (negative ion mode non-targeted lipidomics). See Supplementary Table 4B.

Supplementary Table 4C: Pathway of enrichment analysis under hypoxia in PC3 prostate cancer cells (negative ion mode non-targeted lipidomics). See Supplementary Table 4C.

Supplementary Table 5: List of human primers for qPCR analysis

Gene	Primers	Source	Source ID
**SAT1**	For (5′-TACCACTGCCTGGTTGCAGAAG-3′)<br>Rev (5′-CTTGCCAATCCACGGGTCATAG-3′)	OriGene	6303
**ALOX15B**	For (5′-CAATGCCGAGTTCTCCTTCCATG-3′)<br>Rev (5′-TGATGTGCAGGGTGTATCGGGT-3′)	OriGene	247
**ALOX15**	For (5′-ACCTTCCTGCTCGCCTAGTGTT-3′)<br>Rev (5′-GGCTACAGAGAATGACGTTGGC-3′)	OriGene	246
**LPCAT3**	For (5′-CTTTGGAGCCACCTCTCTACAG-3′)<br>Rev (5′-ACCAGGCTGAAAATGTCTCTTCC-3′)	OriGene	81539
**ACSL4**	For (5′-GCTATCTCCTCAGACACACCGA-3′)<br>Rev (5′-AGGTGCTCCAACTCTGCCAGTA-3′)	OriGene	2182
**GPX4**	For (5′-ACAAGAACGGCTGCGTGGTGAA-3′)<br>Rev (5′-GCCACACACTTGTGGAGCTAGA-3′)	OriGene	2879
**SLC3A2**	For (5′-CCAGAAGGATGATGTCGCTCAG-3′)<br>Rev (5′-GAGTAAGGTCCAGAATGACACGG-3′)	OriGene	6520
**SLC7A11**	For (5′-TCCTGCTTTGGCTCCATGAACG-3′)<br>Rev (5′-AGAGGAGTGTGCTTGCGGACAT-3′)	OriGene	23657
**p53**	For (5′-CCTCAGCATCTTATCCGAGTGG-3′)<br>Rev (5′-TGGATGGTGGTACAGTCAGAGC-3′)	OriGene	7157
**TFRC**	For (5′-ATCGGTTGGTGCCACTGAATGG-3′)<br>Rev (5′-ACAACAGTGGGCTGGCAGAAAC-3′)	OriGene	7037

**DMT1**	For (5′-AGCTCCACCATGACAGGAACCT-3′)<br>Rev (5′-TGGCAATAGAGCGAGTCAGAACC-3′)	OriGene	4891
**SLC39A14**	For (5′-CTGGACCACATGATTCCTCAGC-3′)<br>Rev (5′-AGAGTAGCGGACACCTTTCAGC-3′)	OriGene	23516
**SLC39A8**	For (5′-CCTGCTGTCACAGAAGCTAATGG-3′)<br>Rev (5′-GCAGAGCGTTATCATCCAGGCA-3′)	OriGene	64116
**HO1**	For (5′-CCAGGCAGAGAATGCTGAGTTC-3′)<br>Rev (5′-AAGACTGGGCTCTCCTTGTTGC-3′)	OriGene	3162
**VDAC2**	For (5′-CTTTGCAGTGGGCTACAGGACT-3′)<br>Rev (5′-CGAGTGCAGTTGGTACCTGATG-3′)	OriGene	7417
**VDAC3**	For (5′-CCATAAACCTTGCTTGGACAGCT-3′)<br>Rev (5′-CCAGTCCAATCAGGCTGGCATT-3′)	OriGene	7419
**MAP1LC3B**	For (5′-GAGAAGCAGCTTCCTGTTCTGG-3′)<br>Rev (5′-GTGTCCGTTCACCAACAGGAAG-3′)	OriGene	81631
**NCOA4**	For (5′-GCTTGCTATTGGTGGAGTTCTCC-3′)<br>Rev (5′-GCCATACCTCACGGCTTCTAAG-3′)	OriGene	8031
**NCP1L1**	For (5′-TGCTGTTGTGCAGCCTCTCTGA-3′)<br>Rev (5′-CCACAAAGGCTGACATCTGCAG-3′)	OriGene	29881
**NOX1**	For (5′-GGTTTTACCGCTCCCAGCAGAA-3′)<br>Rev (5′-CTTCCATGCTGAAGCCACGCTT-3′)	OriGene	27035
**GAPDH**	For (5′-CAGCAATGCATCCTGCACC-3′)<br>Rev (5′-TGGACTGTGGTCATGAGCCC-3′)	IDT	
